# Harnessing fungal bio-electricity: a promising path to a cleaner environment

**DOI:** 10.3389/fmicb.2023.1291904

**Published:** 2024-01-30

**Authors:** Aisha Umar, Mustansar Mubeen, Iftikhar Ali, Yasir Iftikhar, Muhammad Aamir Sohail, Ashara Sajid, Ajay Kumar, Manoj Kumar Solanki, Praveen Kumar Divvela, Lei Zhou

**Affiliations:** ^1^State Key Laboratory for Managing Biotic and Chemical Threats to the Quality and Safety of Agro-products, Institute of Agro-Product Safety and Nutrition, Zhejiang Academy of Agricultural Sciences, Hangzhou, China; ^2^Institute of Botany, University of the Punjab, Lahore, Pakistan; ^3^Department of Plant Pathology, College of Agriculture, University of Sargodha, Sargodha, Pakistan; ^4^Department of Genetics and Development, Columbia University Irving Medical Center, New York, NY, United States; ^5^National Key Laboratory of Plant Molecular Genetics, Center for Excellence in Molecular Plant Sciences, Institute of Plant Physiology and Ecology, Chinese Academy of Sciences, Shanghai, China; ^6^Amity Institute of Biotechnology, Amity University, Noida, Uttar Pradesh, India; ^7^Department of Life Sciences and Biological Sciences, IES University, Bhopal, Madhya Pradesh, India; ^8^Plant Cytogenetics and Molecular Biology Group, Faculty of Natural Sciences, Institute of Biology, Biotechnology and Environmental Protection, University of Silesia in Katowice, Katowice, Poland; ^9^Contec Global Agro Limited, Abuja, Nigeria

**Keywords:** catalyst, electricity, environment, fungi, fuel, heavy metals, soil

## Abstract

Integrating fungi into fuel cell systems presents a promising opportunity to address environmental pollution while simultaneously generating energy. This review explores the innovative concept of constructing wetlands as fuel cells for pollutant degradation, offering a practical and eco-friendly solution to pollution challenges. Fungi possess unique capabilities in producing power, fuel, and electricity through metabolic processes, drawing significant interest for applications in remediation and degradation. Limited data exist on fungi’s ability to generate electricity during catalytic reactions involving various enzymes, especially while remediating pollutants. Certain species, such as *Trametes versicolor, Ganoderma lucidum, Galactomyces reessii, Aspergillus* spp., *Kluyveromyce smarxianus*, and *Hansenula anomala*, have been reported to generate electricity at 1200 mW/m^3^, 207 mW/m^2^, 1,163 mW/m^3^, 438 mW/m^3^, 850,000 mW/m^3^, and 2,900 mW/m^3^, respectively. Despite the eco-friendly potential compared to conventional methods, fungi’s role remains largely unexplored. This review delves into fungi’s exceptional potential as fuel cell catalysts, serving as anodic or cathodic agents to mitigate land, air, and water pollutants while simultaneously producing fuel and power. Applications cover a wide range of tasks, and the innovative concept of wetlands designed as fuel cells for pollutant degradation is discussed. Cost-effectiveness may vary depending on specific contexts and applications. Fungal fuel cells (FFCs) offer a versatile and innovative solution to global challenges, addressing the increasing demand for alternative bioenergy production amid population growth and expanding industrial activities. The mechanistic approach of fungal enzymes via microbial combinations and electrochemical fungal systems facilitates the oxidation of organic substrates, oxygen reduction, and ion exchange membrane orchestration of essential reactions. Fungal laccase plays a crucial role in pollutant removal and monitoring environmental contaminants. Fungal consortiums show remarkable potential in fine-tuning FFC performance, impacting both power generation and pollutant degradation. Beyond energy generation, fungal cells effectively remove pollutants. Overall, FFCs present a promising avenue to address energy needs and mitigate pollutants simultaneously.

## 1 Introduction

Rapid global population and industrial growth have led to the depletion of fossil fuels to meet the increasing demand for energy generation. The exploration of efficient and innovative approaches has captured the interest of environmental researchers seeking to address and remediate ecological pollutants. Fungi have demonstrated the ability to generate power using biodegradable waste, reducing the need for conversion (Wu et al., 2020). Conventional methods face limitations such as extensive land requirements, high capital costs, and complex production procedures. Continuous bioenergy generation offers a sustainable alternative to non-renewable power sources (Idris et al., 2023) because sustainable bioenergy resources are increasing worldwide as an alternative to conventional or chemical methods for power generation. Biological degradation involves, for some, using microorganisms (fungi, algae, bacteria, and enzymes) and is better than other biological methods (e.g., plants), which utilize a large land area, exhibit very high sensitivity toward toxic dyes, and require a long consumption time (Ameen et al., 2020; Umar et al., 2023).

Fungal species have been recognized for their capacity as “novel cell factories” in energy production (Mandal et al., 2023). Fungal activity significantly influences the degradation rate of recalcitrant compounds (Vaksmaa et al., 2023). Fungal fuel cells (FFC) represent a technology that harnesses biodegradable waste materials in the production of power during the treatment of contaminated surfaces (sediments, soil, and wastewater). Some scientists have utilized this innovation to generate electricity, relying on electrodes combined with fungi to degrade toxic waste products (Franks and Nevin, 2010). Well-known fungal species can generate power by breaking down waste materials through complex enzymatic systems (Khan et al., 2023). Fungal species have gained recognition for their unique role in bioremediation (Maldonado et al., 2023). Fungal electrochemical technology (FET) focuses on generating energy through pollutant mitigation (substrates), a topic of this review article. Fuel cells based on microbes, algae, bacterial and fungal cultures also have applications in the field of fuel.

The significant demand for petroleum products leads to environmental problems, e.g., pollution and global warming. The demand for renewable energy sources, such as fungal cell factories, has grown due to the limited resources of fossil power and the escalating issues related to global warming (Naaz et al., 2023; Ameen and Al-Masri, 2023a). These processes are particularly effective for remediating hazardous recalcitrant materials and toxic organic pollutants (Malik et al., 2023). Various types of industrial equipment, both small and large, are now being used across various industries. Biological degradation involves microorganisms like fungi, algae, bacteria, and enzymes, which are sensitive to toxicity and require significant land area and time (Flimban et al., 2019; Ahamad et al., 2023). Energy-producing fungal biocatalysts enhance electron transfer through extensive hyphal networks, generating stable electricity through “external electrochemical operations” (Hanafi and Sapawe, 2020). Yeasts and fungi are considered more crucial than bacteria due to their unique feature (Sayed and Abdelkareem, 2017); as fungi break down organic materials, they release electrons necessary for electricity generation.

Fungal cells are known for their capacity for high-quality biofuel production, bioelectricity generation, and pollutant treatment (Ahmad et al., 2023). Utilizing the metabolic activities of fungi, microbial fuel cells can efficiently break down organic contaminants and waste from agriculture into simpler components, effectively mitigating the environmental impact of hazardous compounds (Al-Masri et al., 2023; Alwakeel et al., 2023). This review primarily focuses on FFC as a viable technology for reducing environmental pollutants, while simultaneously producing electricity. Biotic sources utilize various fungal species, but limited data is available on energy production using electrochemical systems involving fungi (Al-Sabri et al., 2014; Mehta et al., 2023). This review will delve into different fungal species used in biological fuel, techniques for cultivating and preserving fungal cultures, and their application in various industries for bioremediation, biodegradation, and bioenergy production. Fungal fuel cells offer a sustainable and effective approach to environmental contamination treatment by combining pollutant remediation with power generation, contributing to a greener and more sustainable future.

## 2 Construction of fungal-mediated fuel cells

FFCs operate based on the principles of oxidation–reduction reactions in anodic and cathodic regions, which take place through a network of “microbial and electrochemical pathways” (Slate et al., 2019).

- Anodic compartment

In the anodic compartment, protons and electrons are produced through the oxidation of substrates by fungal species, Electrons and protons are generated by the oxidation of organic material in the aqueous solution in the anodic chamber, where fungal catalysts are employed. These extracellular species, known as “exoelectrogens” (biocatalysts), facilitate electron transfer (He et al., 2017). Biochemical reactions result in stable reduced products through the interaction of H^+^ and e^−^ (Mittal et al., 2023). Fungi can transfer the e^−^ to the anode through three potential pathways: (1) pili/conductive wires; (2) direct contact; (3) redox mediators/electron shuttles (Kumar et al., 2016). The anode fungus facilitates electron transfer through “redox-active” synthetic mediators and fungal proteins. Specific microorganisms, like *Shewanella oneidensis* and the hyphal circuits of *T. versicolor*, enable efficient e^−^ transfer, enhancing the degradation of various biological substrates. The use of fungal cells to generate electrical energy dates back to 1911 (Potter, 1911).

- Proton exchange membrane

A “proton exchange membrane (PEM)” is used to separate the cell into anodic and cathodic chambers (Kim I. S. et al., 2008; Kim J. R. et al., 2008). The membrane serves the purpose of segregating anolyte and catholyte to prevent intermixing between the two compartments and to minimize the presence of oxygen in the anolyte.

- Cathodic compartment

**Figure 1:**
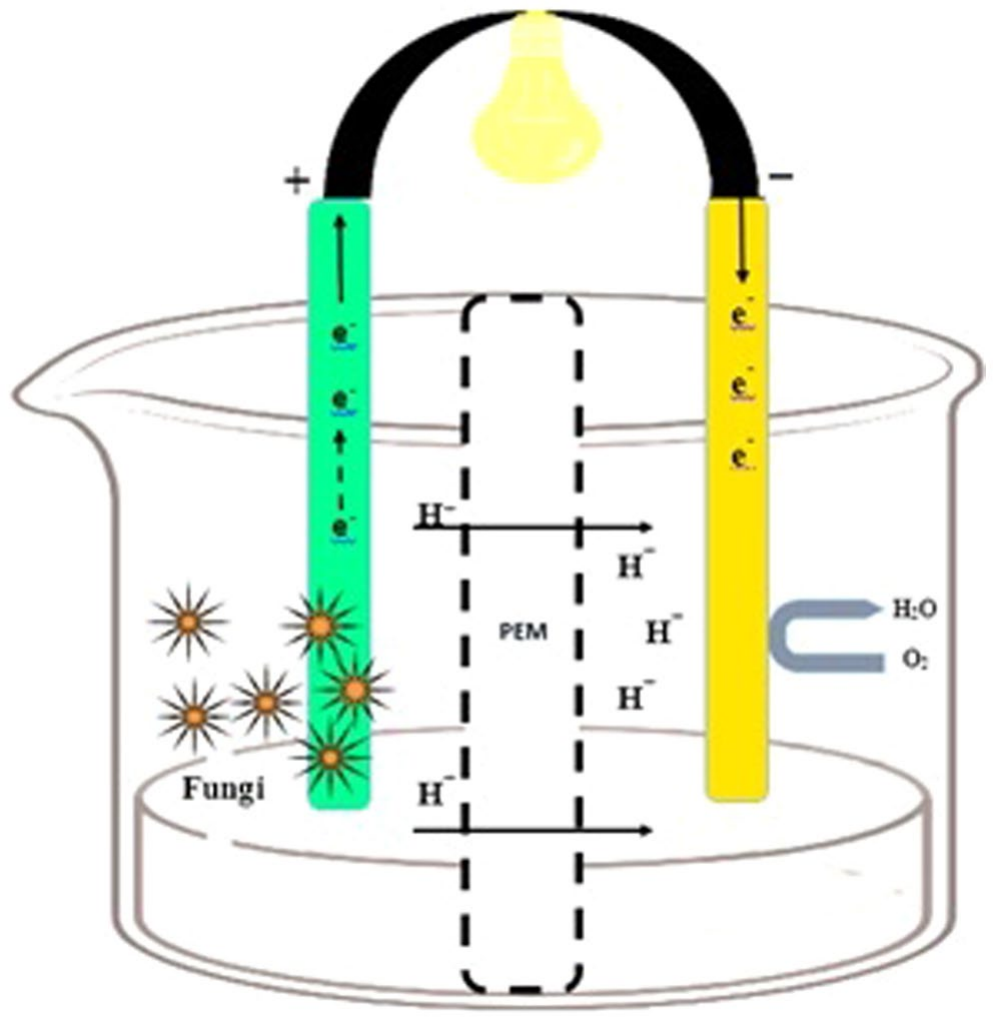
Design of the fungal fuel cell. An anodic chamber containing fungal species and substrate for degradation (left side) and a cathodic chamber for proton and O2 acceptance (right side).

In the cathodic chamber, oxygen is reduced to water by accepting the terminal electrons coming from the anodic region. The proton exchange membrane (PEM) in the cathodic compartment facilitates proton diffusion (Miskan et al., 2016). Protons and electrons move toward the cathodic chamber through PEM, and their combination (H^+^ and e^−^) generates water molecules. Fungi enzymes act as a catalyst for the reaction, and the concluding electron and proton recipient is oxygen (Figure 1). Furthermore, it is imperative to have a hermetically sealed compartment that offers sufficient room for the placement of electrodes, inlets, and outlets. This is crucial for the purpose of effectively arranging the electrodes and PEM within the entirety of the system (Abubackar et al., 2023).

### 2.1 Types of electrodes

The selection of electrode materials for FFCs plays a crucial role in determining their performance. As observed by Mustakeem (2015), the choice of electrode material directly impacts electrode kinetics. The materials used for the cathode (Platinum/Nafion) and anode (carbon cloth, carbon paper) are delicate and expensive. FFCs with fungal components offer cost-effective electrodes, high power output, and a versatile membrane suitable for treating various effluents, e.g., resizing, dyeing, bleaching, and printing. The selection of appropriate electrode material is a critical factor in optimizing the efficiency and effectiveness of FFCs in various applications, including electricity generation and environmental pollutant remediation. Selecting the appropriate electrode material is an essential aspect of optimizing FFC performance and achieving desired outcomes in sustainable energy production and pollution control.

#### 2.1.1 Oxidation reaction

Anodes made of carbon (carbon paper, carbon cloth, carbon nanotubes, carbon felt) are non-corrosive and cost-effective; however, stainless steel, graphene, gold, and titanium can also be utilized for the same purposes (Richter et al., 2008). These materials enhance the anode’s surface properties, providing an ideal platform for fungal catalysis. The quality of the anode surface is critical, improving its surface area, biocompatibility, electrical stability, and FFC efficiency (Watanabe, 2008). Anode materials significantly impact performance, acting as the driving force for power generation. Several yeast strains, including *Saccharomyces cerevisiae*, *Candida melibiosica*, *Hansenula polymorpha*, *Pichia anomala,* and *Blastobotrys adeninivorans* in the anodic chamber, are known for their ability to generate electricity (Prasad et al., 2007; Gunawardena et al., 2008; Haslett et al., 2011; Shkil et al., 2011; Hubenova and Mitov, 2015).


$$\begin{array}{l} \mathrm{Oxidation Reaction}\;\mathrm{at}\;\mathrm{Anode}=\\ C_{6}H_{12}O_{6}+6H_{2}O\to 6CO_{2}+24H^{+}+24e^{-} \end{array}$$

There are several advantages to FFCs. The cost and power output limitations (Zhang et al., 2017) can be overcome by selecting the appropriate anode materials with a broad surface area, super hydrophilicity, excellent chemical stability, high porosity, high electrical conductivity, and biocompatibility, which improve the electron transfer (Franks and Nevin, 2010; Santoro et al., 2015). The hydrophobic nature of the anode negatively affects microbial adhesion, increasing resistance to electron transfer and reducing current density and power (Xu et al., 2018). Iron oxide nanoparticles, iron, carbon cloth, and graphite carbon felt serve as effective catalysts for the anode, enhancing the treatment of industrial pollutants (Mohanakrishna et al., 2018).

#### 2.1.2 Reduction reaction

Various substrate types act as the cathodes in FFCs (sewage wastage rich in organics like sucrose, lignocellulose, glucose, biomass materials, acetate, and biodegradable waste such as brewery waste, etc.). Oxygen reduction occurs in the cathodic chamber (Jadhav et al., 2014). Electrons from the anode reach the cathodic chamber via an external network, and protons are transferred through the PEM. The cathode significantly influences the voltage output and exhibits “large redox capability.”


$$\mathrm{Reduction}\;\mathrm{Reaction}\;\mathrm{at}\;\mathrm{Cathode}=6O_{2}+24H^{+}+24e^{-}\to 12H_{2}O$$

A biocathode, composed of less costly, stable, and non-chemical components, is employed in FFCs today due to its various biological compartments in the cathodic electrode. Fungi are placed in an oxygenated cathode.

## 3 Fungal characteristics in fuel cell

The remarkable performance of FFC is influenced by co-inoculation or consortium of different fungal species (Guo et al., 2014). Both antagonistic (reduction) and synergistic (enhancement) relationships are possible among different species for power density and remediation of pollutants. Fungal biocatalysts influence the total internal resistance, degradation of organic pollutants, and transportation of e^−^ toward the anode (Hodgson et al., 2016). FFCs are easy to operate under ambient environmental conditions, producing the minimum amount of sludge (You et al., 2006). Certain enzymes are affected by electric fields. The permeability of cell membranes increases the efficiency of the removal of OC and absorption of extracellular substances, respectively. This, overall, promotes the metabolism rate of fungi. Fungi play a dual role in FFCs; for example, fungi support electron transfer at the anode through chemical mediators or respiratory proteins (Shabani et al., 2021). Maximum internal resistance, solution condition, and slow kinetics of enzymes lead to lower power density (He et al., 2017). The degradation rate depends on the fungal metabolism rate and molecular weight. Low molecular weight compounds are mineralized/degraded faster than higher weight (Zhou et al., 2020). The maximum or minimum temperature increases the fungal metabolism (depending on the fungal species) and membrane permeability, which enhances the output of FFCs (Oliveira et al., 2013).

## 4 Diesel production and electricity generation

### 4.1 Fungal biocatalytic action in diesel production

Fungal fuel cells appear to be very helpful. Biofuel comprises bioethanol, biohydrogen, and biodiesel production. Catalysts enhance the biodiesel generation. During the treatment of pollutants with enzymes, the carbon substances of lower-chain produce are utilized for oxidation. Exoelectrogens are examined for the growth of the FFCs that cause the change of the organic material into electricity, ethanol, and H_2_ gas (Patil et al., 2012). Biocatalysts act as “exoelectrogens” that oxidize the organic compounds and transport electrons from the anodic chamber to the cathode, along with electricity generation. Biocatalysts are microorganisms deposited onto a carbon-based anode as a floating biomass in the yeast FFC (Christwardana et al., 2018). Yeast FFCs benefit from disintegrating complex broader substrates (substrates that depend upon cellulose and starch into organic substances) (Mao and Verwoerd, 2013). Oleaginous fungal species have great potential for producing biodiesel. It represents a substitute for renewable energy, and the production of fatty acids (oils) (Ratledge, 2004). Some oleaginous species catalyze xylose and help form lipids from “lignocellulosic hydrolysates” (Kurosawa et al., 2013).

Genera like *Mucor* and *Aspergillus* store the oils in cells. Those strains have huge lipid contents and metabolize the TAG (triacylglycerides) to produce biofuels. Zygomycetes are the best oleaginous species, which provide oleic acids and palmitic for biodiesel formation. These fungi also facilitate the degradation of biogas from specific biomass (Dhanasekaran et al., 2017). Hydrolytic and lignolytic fungi have recently reported the production of bioethanol or biofuels (Robak and Balcerek, 2018). Some Basidiomycetes are reported to secrete extracellular enzymes that break down waste materials and produce electricity with fuels (Beopoulos and Nicaud, 2012). The famous species *Colletotrichum* spp., *Saccharomyces cerevisiae, Candida* spp., *Alternaria* spp., *Penicillium* spp., *Yarrowia lipolytica, Rhodosporidium toruloides, Cryptococcus* spp., *Trichoderma reesei*, *Aspergillu*s spp., and *Rhizopus* show a capacity for the production of biofuel and the generation of electricity during electron transmission through cytochrome C (Sekrecka-Belniak and Toczyłowska-Mamińska, 2018). Pure *Saccharomyces cerevisiae* is ideal in FFCs. Christwardana et al. (2018) demonstrated the importance of yeast in MFCs due to its characteristics. It is a non-pathogenic species to non-targeted individuals with a considerable growth rate. It can easily be cultured in anaerobic environmental conditions and grows well at room temperature to effectively treat pollutants (Hubenova and Mitov, 2015). Low cost, swift multiplication, activeness, and survival in a dry environment make this species suitable for fuel cells.

Carbon-neutral fuel, “Bioethanol,” is collected from yeast (fungi), plants, and wasted bacteria/algal biomass material (Hanaki and Pereira, 2018). Yeasts like *Kluyveromyces marxianus, Candida melibiosica, Blastobotrys adeninivorans, P. polymorpha, Hansenula polymorpha, Pichia anomala*, and *Saccharomyces cerevisiae* with their biocatalysts are used in the FFCs without an outside mediator (Haslett et al., 2011; Shkil et al., 2011). *Kluyveromyces marxianus,* a promising yeast, produces maximum power under high temperatures when grown in natural substrates (organic)*. Trametes versicolor, Pleurotus ostreatus*, and *Ganoderma lucidum* are prominent mushrooms showing better electricity output. *Trametes versicolor* with glucose is used to produce bioenergy through its enzymatic system. Fernandez de Dios et al. (2013) examined the fungus’s role in FFCs for energy production through pollutant degradation. Microbes *Shewanella oneidensis* and *T. versicolor* have a 30-day biofilm development period and accelerate substrate degradation by enhancing anode electron transport.

### 4.2 Role of exogenous mediators in electricity generation

**Figure 2:**
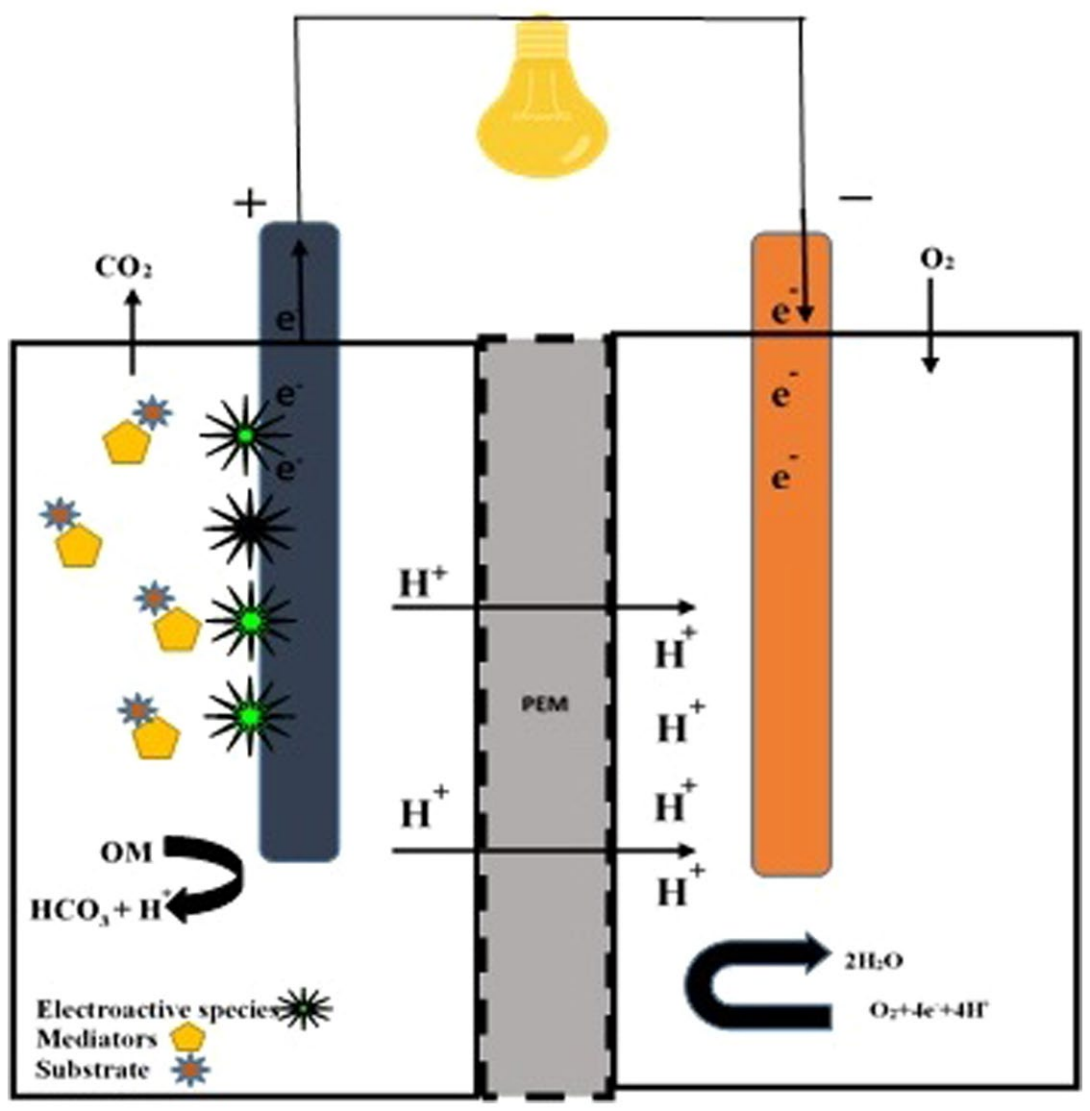
Double-chambered fungal fuel cell. A double-chambered fungal fuel cell equipped with electroactive fungal species, mediators, and substrate. Proton transfer to the cathode is facilitated by a proton exchange membrane (PEM).

Non-living exogenous mediators improve electricity power with the support of fungal species. Methylene blue (MB), neutral red (NR), methyl (yellow, red, orange), bromophenol blue (BPB), bromothymol blue (BTB), bromocresol green (BCG), bromocresol purple eosin, cresol red (CR), murexide, and eriochrome black T are used to enhance electron transport between microbes and the anodic chamber. In non-mediator FCs, *S. cerevisiae* caused the electrons to move to the anodic chamber via confined species (Sayed et al., 2012). Babanova et al. (2011) explained that exogenous mediators play an active role in the kinetics of electron transfer and simultaneously reduce cell catabolism. White rot fungus oxidized ABTS at 420 nm and indicated the construction of a fungal fuel cell (Figure 2). *Pichia anomala* acts as a biological catalyst (ferricyanide reductase and lactate dehydrogenase) along with glucose molecules to generate electricity (Prasad et al., 2007). Its cells showed covalent connections or physical means. *Blastobotrys adeninivorans* is a dimorphic yeast. The species’ biocatalytic action in a dual-chambered cell recorded a peak energy density of 28 mWm − 2 (Haslett et al., 2011). FC centered on *B. adeninivorans* exposed better results than *S. cerevisiae*. In a mediator-less dual chamber FC, the catalytic action of *Schizosaccharomyces pombe* and *C. glabrata* is significant (Kaneshiro et al., 2014).

## 5 Fungal catalytic role in FCs

Fungal mycelia grow on the surface of the anode or in the anodic chamber, releasing their degradative and fermentative enzymes. The traditional biological procedure is insufficient in pollutant remediation because of the absence of “biocatalytic activity” (Dawoud et al., 2021; Al-Enazi et al., 2023a,b). Deposited catalysts have gained significant interest at electrodes, accelerating the kinetics reaction (the electrodes). Cathodic catalysts increase the number of reduction reactions. Thus, an anodic catalyst increases the rate of oxidation reaction. Biocatalysts provide a sustainable, clean, and renewable energy source using exoelectrogenic microbes (Chae et al., 2009). Oxidoreductase is famous for disintegrating numerous pollutants (Rauf and Salman, 2012). Reactive diffusible redox-based mediators (oxidoreductase) drastically enhance the reaction rate, which is broken down by the enzymes (Adelaja et al., 2015; Ameen and Majrashi, 2023b). Many systems of enzymes are successful in degrading many kinds of pollutants by oxidative breakdown into smaller molecules. The benefits of enzymatic degradation are the small number of reagents under mild states and the degradation of various substrates. The disadvantage is the significant cost of the enzymes, which could be improved via a technology that is recombinant DNA. Ferricyanide reductase and Lactate dehydrogenase are readable enzymes (Prasad et al., 2007; Al-Enazi et al., 2023a,b). Wood degraders secrete many extracellular enzymes, such as laccase. Laccase is manufactured when a fungus causes the breakdown of the lignin compound (huge polymers) which are more common in the natural habitats of the white rot fungus (Kadir et al., 2018). Fungal laccase acts as a 4Cu that contains the oxidoreductase-based bio-catalyst, which moves the electrons in the fuel cell. Laccase has been found to transfer noble metals (Mani and Hameed, 2019; Nelagadarnahalli et al., 2023).

No cathodic chamber is found in a single-chamber FC; its cathode is directly exposed to air for maximum O_2_ availability (Crini et al., 2019). Laccase produces fungi that cause white rot on the cathode (Shleev et al., 2005). Finally, this laccase behaves as a cathodic chamber and acts as a catalyst in a fuel cell of fungi (FFC). The fungi cause white rot to metabolize laccase for the nutrients’ acceleration by the breakdown of the lignin compound. These are cost-effective and suitable for the sustainable development of power generation by *in situ* elimination of the laccase. The fungi causing the white rot enhances the efficacy of those FFCs. The species of *Ganoderma lucidum* BCRC 36123, which have the ability to produce laccase, were positioned on the cathode surface within the FC compartment. This placement resulted in the degradation of the dye within the anodic chamber, in addition to the presence of anaerobic microorganisms. Laccase on biocathode minimizes the cost while manufacturing the FFCs. The activity of the Laccase (1,063 ± 26 U/L) of fungi causing the disease white rot (*Phanerochaete chrysosporium, Pycnoporus cinnabarinus, Ceriporiopsis subvermispora,* and *Trametes pubescens*) removed 87–92% of phenolic materials at pH 5.0 (Robinson et al., 2001; Strong, 2010). Laccase fungi hydrolyzed the winery wastewater and potentially removed the phenolic compounds, color, and COD (Strong and Burgess, 2008; Boutafda et al., 2023). The renowned fungal species *Pleurotus ostreatus* and *T. versicolor*, which are known for their ability to produce laccase, generate energy via the enzymatic layer located within the fungi’s cathode electrode (Mani and Hameed, 2019). Lignin degrades as the laccase generates. When lignin in plants breaks down in the soil, laccase replaces nutrients, producing tremendous power generation in two ways (Wu et al., 2012). Ligninolytic is an extracellular oxidative enzyme that allows fungi to break down contaminants and xenobiotic materials (Wesenberg et al., 2003). There is little research on PPCP degradation (Wang et al., 2012). Pollutants that cause the breakdown of the peroxidases include heme-peroxidases and non-heme peroxidases and they can be classified into four superfamilies: peroxidase peroxygenase, peroxidase chlorite dismutase, peroxidase-catalase, and peroxidase cyclooxygenase (Zamocky et al., 2008). Phytase is an active fungal catalyst. *Candida melibiosica* has potential phytase action in the dual compartment under carbon sources (glucose, fructose, and starch) (Hubenova et al., 2014). This type of fuel cell generates 60 mWm^3^ bioelectricity without an extracellular mediator at the expense of fructose substrate (Hubenova et al., 2014). It opens up a novel avenue to utilize non-chemical and sustainable approaches for the cleanliness of phosphate-polluted water and the degradation of many xenobiotic contaminants. Enzymes catalyze the toxic pollutant material into the phase, which is significantly less harmful. The by-products are produced to minimize the contaminants of air, soil, and water (Mohana et al., 2013). Fungal species that secreted the enzymes to mitigate pollutants in fuel cells are listed in Table 1.

**Table 1:** Enzymes catalytic action for mitigation of pollutants in FFCs.

Extracellular enzyme	Action	Pollutants	References
Laccases	Oxidations	(PAH, phenolic azo dyes, phenol and chlorinated phenol, TNT excreted metabolites)	Nyanhongo et al. (2006)
Manganese peroxidase	Oxidations	(PAH, different types of dyes, TNT excreted metabolites)	Qin et al. (2014)
Hydroquinone quinone	Peroxidase reactions to produce Fenton reagent	2-fluorophenol	Kramer et al. (2004)
Hydroxyl radical	Hydroxyl attack and oxidation	(Hydroxylation of chlorinated hydrocarbons)	Marco-Urrea et al. (2010)
Intracellular enzyme			
Transferases	Removal of OH groups to produce conjugates	Secrete conjugates	Hundt et al. (2000)
Oxidases	Epoxidations,	PAH, dioxins, pharmaceuticals, and herbicides	Hata et al. (2010)
Oxidases	Hydroxylation	*P. chrysosporium* encoding for 150 cytochrome P450	Kasai et al. (2010)
Quinone reductases	Production of OH radicals via Fenton reaction	Enabling extracellular pollutant attack	Jensen et al. (2001)
Aromatic nitro reductases	Reductions of nitro groups for further extracellular degradation	Nitro group reduction in 1,3-dinitrobenzene, 2,4-dinitrotoluene, 2,4,6-trinitrotoluene, 1-chloro-2,4- dinitrobenzene, and 2,4-dichloro-1-nitrobenzene, TNT reduction to hydroxylamine- and dinitrotoluene	Esteve-Núñez et al. (2001)

## 6 Biological method and mechanism of degradation

Fungal degradation is renowned for pollutant treatment by consuming substrates. Yeast cells metabolize hazardous compounds in water, soil, and air and can be categorized into two types: oxidized and fermented (Rozene et al., 2021). Fermented yeast converts 6C sugar into CO2 and alcohol. Yeast glycolysis transforms glucose into pyruvate, involving glyceraldehyde oxidation, yielding glycerol, and phosphoenolpyruvate. Under aerobic conditions, decarboxylation generates acetaldehyde, converted into acetic acid. Alcohol is released anaerobically (Li et al., 2015). If glycolysis pyruvate undergoes oxidative decarboxylation before entering the Krebs cycle, it becomes acetyl-CoA, releasing CO2 and contributing to acetic acid production. This oxidation releases electrons, decreasing the number of carbon atoms and transforming acetate into acetyl-CoA for the Krebs cycle. Electron Transfer Chain activation uses high-energy molecules for redox reactions (Christwardana et al., 2021).

**Figure 3:**
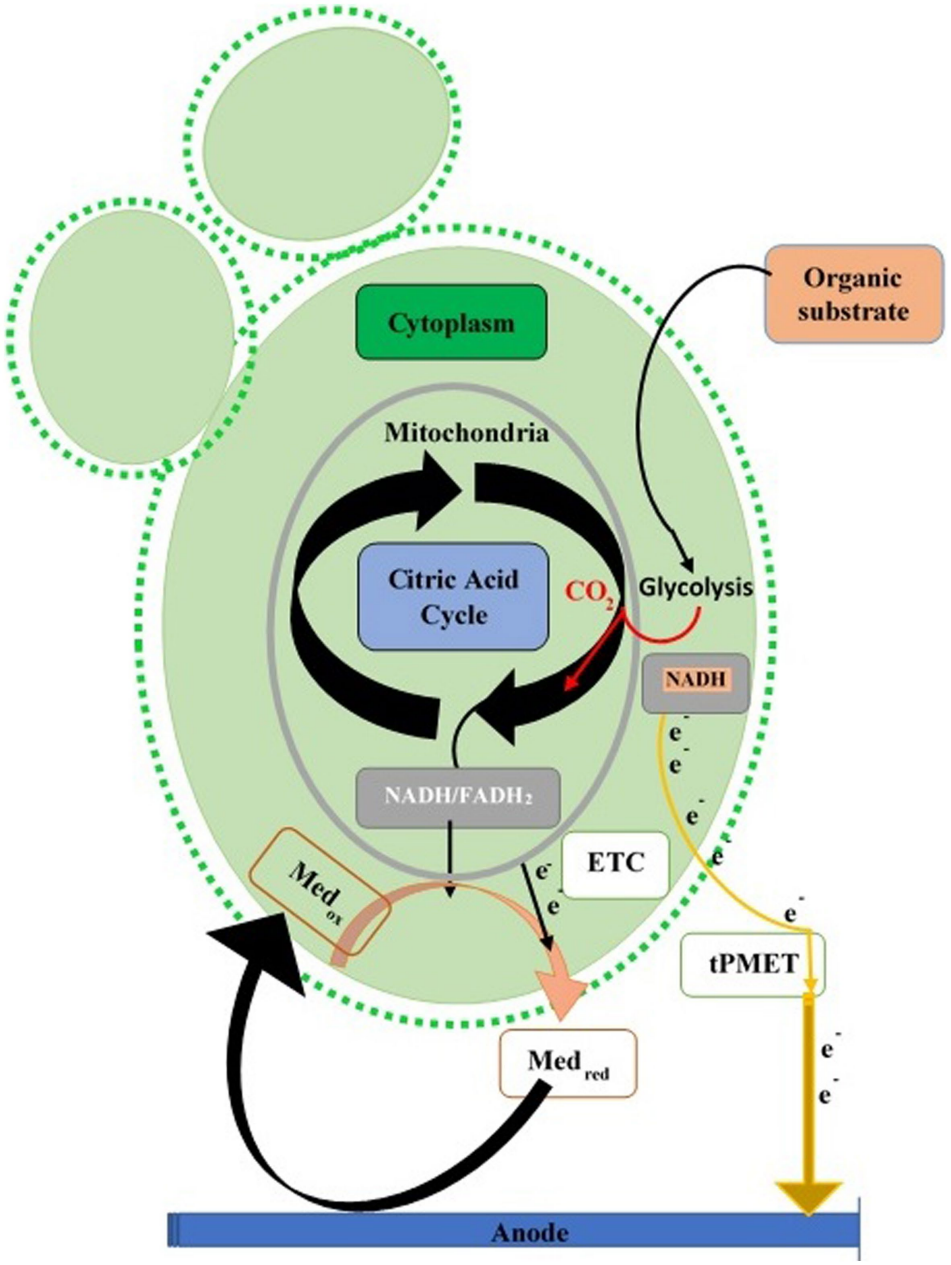
Extracellular electron transfer paths in yeasts. Electron transport chain (ETC) of mitochondrion transfers the electron (direct or mediated) toward the anode of the fuel cell via redox mediators or trans-Plasma Membrane Electron Transport (tPMET).

Yeast cell walls, thick with a trans-plasma membrane electron system or “plasma membrane (PM) oxidoreductase,” transfer electrons from cytoplasmic molecules (NADH and NADPH) to an external acceptor, used in reducing Fe2+ to Fe3+ or ATP production (Lesuisse and Labbe, 1992). Electron exit via this route is small compared to total electrons from aerobic catabolism. Yeast fuel cells play a vital role in glucose oxidation for energy (Gunawardena et al., 2015). Glycolysis increases in some yeasts during anaerobic conditions, compensating for mitochondrial ATP loss. The trans-plasma membrane mechanism transfers electrons during anaerobic anode, increasing cytoplasmic NADH/NADPH. Hydrophilic mediators cross the membrane via trans-plasma membrane proteins, while lipophilic mediators access internal reduced molecules (NADH and NADPH) and mitochondria (Walker, 1998; Figure 3).

### 6.1 Algae and fungus co-culture

Algae, in conjunction with fungi, are excellent for FCs to generate electricity. Protons migrate from yeast to algae and are transported via ‘microporous tubes’ embedded in the active bleached earth known as “ion exchange medium” for electricity generation. This facilitates the transfer of extracellular electrons, advancing fuel cell technologies. Genetically modified and natural yeast with enhanced enzymatic activity aid in the degradation of toxic substances and electricity production. Filamentous fungi aggregate into a pellet in a submerged culture. The process is divided into 3 phases (Espinosa-Ortiz et al., 2016). Micro-morphological growth – Phase 1: here, fungal spores swell and germinate to form embryonic mycelium (Espinosa-Ortiz et al., 2016). Macro-morphological growth – Phase 2: mycelium starts to branch, subsequently, and convolute into visible pellets (accompanied by hyphae-to-hyphae interactions). Pellet self-decomposition Phase 3: deterioration of growth conditions, the hyphae begin to self-dissolve.

**Figure 4:**
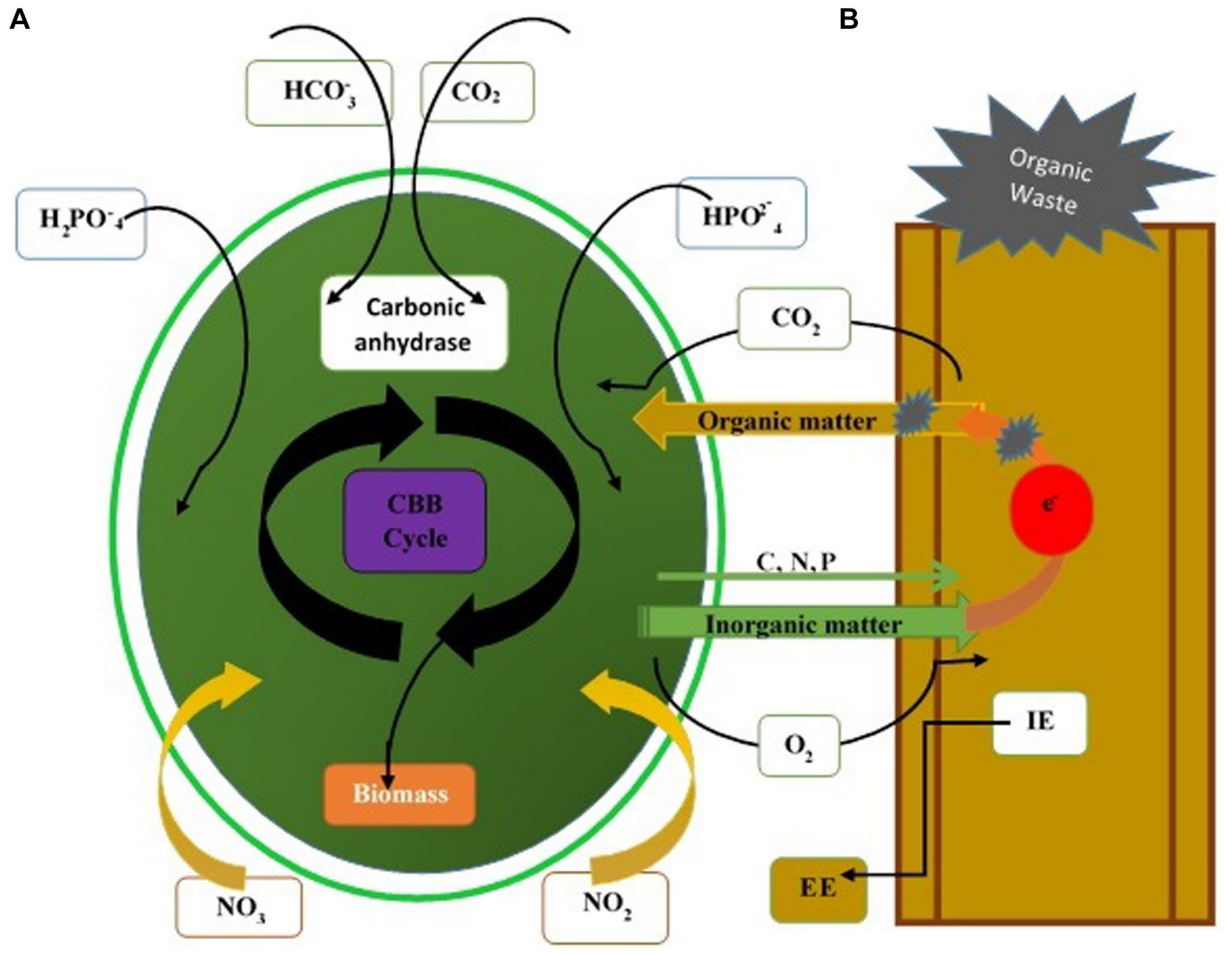
Mechanisms of pollutant removal through the fungal-microalgae consortium. **(A)** Adsorption or capture of suspended solid particles. Degradation by extracellular enzymes (EE) and intracellular enzymes secreted by fungi. **(B)** Assimilation of soluble nutrients by microalgae and fungi. CBB, Calvin-Benson-Bassham cycle.

Fungi assist microalgae flocculation, a popular method in recent years due to its low cost and high efficiency. Microalgae and fungi form fungi-microalgae pellets under optimum co-cultured conditions. Co-pelletization of fungi and microalgae may interact with the microalgae cells and fungal spores at any stage of palletization. Microalgae cells bind to fungal cells rather than entrapping within the fungal hypha (Wrede et al., 2014). At neutral pH, algae have a negative surface charge due to the presence of functional groups (hydroxyl or amine group). Positively charged fungi serve as a cationic flocculant to neutralize the negative charge on the microalgae and thus adsorb the microalgae cells (Wrede et al., 2014). Yeast and fungus efficiently decolorize the various organic compounds (Thulasinathan et al., 2022). The biodegradable potassium ferricyanide, organic wastewater, and acetic acid are utilized as an anolyte feed. Yeast is renowned for producing various enzymes, lipids, and glycolipids used in the remediation of wastes containing a high concentration of heavy metals, organic matter, and domestic sewage. The high concentration of organic matter is rapidly disintegrated through the yeast consortium (Mohammed et al., 2023). Removal of maximum COD from wastes is achievable in fungi (heterotrophic)-microalgae (mixotrophic) growth systems. Free CO_2_ diffuses into microalgae cells and enters the “Calvin-Benson-Bassham” cycle via rubisco or other enzymes, producing O_2_ and other organic matter for their metabolisms. CO_2_ in the form of bicarbonate in water and carbonic anhydrase of microalgae absorb carbonate or convert it into free CO_2_ directly (Gonçalves et al., 2017; Figure 4).

### 6.2 Synergistic effects

fungi-microalgae co-culture is higher due to the high proportion of fungal biomass. The synergy between fungi and microalgae promotes one another’s growth. Nutrient sharing and gas exchange enhance the individual’s metabolic activity, and fungi can provide shelter to microalgae (like lichens) (Piercey-Normore and Athukorala, 2017). Therefore, the consortium can accumulate nutrients from the surroundings. Fungi use the carbon resources stored in the cell walls of microalgae due to different secretions of extracellular enzymes during their growth. Shading consequence usually restricts the growth of autotrophic microalgae when the microalgae biomass reaches a specific concentration in ponds that barely get any sunlight. Suspended microalgae cells are voluminously fixed in fungal pellets, which facilitates light transmission, growth, and yield of microalgae and significantly increases the algal biomass yield. The decolorization of wastewater by fungi may have the same effect on promoting light transmission.

### 6.3 Yeast metabolizes water, soil, and air pollutants

Industrial wastewater contains various dye categories, including acidic, basic, anthraquinone, and azo dyes (Langhals, 2004). Human consumption releases these pollutants into ground and surface water (Burkina et al., 2015). Biological techniques are environmentally friendly, cost-effective, and receive considerable attention for removing and managing harmful compounds from wastewater bodies (Chaalal et al., 2015; Liu et al., 2015). The degradation of several chemicals from polluted water was carried out by yeast and yeast-like fungi, which included indigo dye (Bankole et al., 2017), methylene blue (Eltarahony et al., 2021), bisphenol A (Neamţu and Frimmel, 2006), and dichlorodiphenyltrichloroethane (DDT) by yeast *Pichia kluyveri* FM012 (Isia et al., 2019), methylene blue by yeast-bacteria consortium (Eltarahony et al., 2021), indigo dye (Bankole et al., 2017) from polluted water. Yeast strains and yeast-like fungi degrade the hydrocarbon from petroleum and oil-contaminated wastewater (Farag and Soliman, 2011; Gargouri et al., 2015). Yarrowia lipolytica PG-20 and PG-32, oil-degrading yeast strains from the Persian Gulf (Hassanshahian et al., 2012). Aromatic structures in wastewater are also biotransformed and detoxified by yeasts (Schlüter and Schauer, 2017).

Yeast fungal species effectively metabolized the petroleum-polluted soils containing hydrocarbon (n- and isoalkanes, isoprenoids) (Chaillan et al., 2004). The polyester polyurethane plastics inside the soil include polyhydroxyalkanoates, polylactic acid, polycaprolactone, polyester polyurethane, and polyvinyl chloride. *G. pannorum* was responsible for the degradation of plasticized polyvinyl chloride (pPVC) (Barratt et al., 2003). Sabev et al. (2006) and Cosgrove et al. (2007) reported that pPVC played a significant role in the degradation of PVC in Bulgarian grassland soil. Poly (butylene succinate-co-adipate) (Yamamoto-Tamura et al., 2020), aliphatic hydrocarbons (Daccò et al., 2020), and high-molecular-weight polycyclic aromatic of the soil was effectively degraded by yeast fungal species (Boonchan et al., 2000). Fungal biocatalysts effectively degrade styrene, toluene, α-pinene, benzene, alkylbenzenes, and other related volatile organic pollutants for air pollution control. Filamentous and yeast-like fungi metabolize volatile pollutants, e.g., as non-oxygenated aromatic pollutants (benzene, alkylbenzenes, styrene), and other related compounds (Kennes and Veiga, 2004). Alkyl- and alkenyl-benzenes are also important air pollutants, and the recent removal of those compounds by fungi is very popular (Estévez et al., 2004). Hydrophobic compounds from polluted air by inoculation in fungal-based bioreactors or yeast-like fungi were it was started just over ten years ago to produce hydrophobic chemicals from contaminated air by inoculating yeast-like fungi or fungal-based bioreactors (Cox et al., 1997).

## 7 Mitigation of pollutants

A modern society cannot function without chemicals. However, the use of chemicals in industries such as leather, pharmaceuticals, pulp, and paper results in environmental pollution and associated negative impacts. The potential of fuel cells lies in their ability to remove pollutants. The removal of pollutants can be measured by organic removal, which is equivalent to the COD between the influent and effluent (Pant et al., 2010). The advantages include the requirement of low concentrations of reagents for the gentle and broad degradation of substrates. Biodegradable organic materials range from pure compounds like glucose, cysteine, acetate, and ethanol to mixtures of organic compounds found in landfills leachate, animal waste, agricultural waste, and industrial liquid waste (Pant et al., 2010).

### 7.1 Mitigation of aromatic organic pollutants

Aromatic organic compounds are carcinogenic and pose serious health hazards (Martins et al., 2015). Organic contaminants serve as preferable substrates for bacterial growth, which reduces oxygen levels in water bodies, increases turbidity and color, and decreases the photosynthetic biota in water (Molla et al., 2023). Polycyclic aromatic hydrocarbons are highly toxic, mutagenic, teratogenic, carcinogenic, genotoxicity, and immunotoxicogenic to various life forms (Bolden et al., 2017). Acute health effects of aromatic compounds include eye irritation, diarrhea, vomiting, skin irritation, confusion, and inflammation (Abdel-Shafy and Mansour, 2016). Naphthalene, benzo(a)pyrene, and anthracene directly irritate the skin and skin sensitizers for animals and humans (Rengarajan et al., 2015). Chronic health effects, e.g., eye cataracts, kidney and liver damage, breathing problems, lung malfunctions, decreased immune function, and asthma-like symptoms are also caused by aromatic HCs. Naphthalene breaks down the red blood cells if ingested or inhaled in large amounts (Rengarajan et al., 2015). Various techniques, including physical, biological, and chemical methods, are employed to treat aromatic organic pollutants. Limited studies are reported on the mechanisms and pathways in the breakdown of PAHs via mycoremediation (Aydin et al., 2017; Agrawal et al., 2018). Direct fungi application in the field has many limitations including inadequate biomass growth, huge biomass handling difficulties, lack of application methodologies, and bulk degrading enzyme production, which can be overcome by oxidative fungal enzyme-mediated PAH bioremediation (Harms et al., 2011; Zhao et al., 2019).

Fungal laccase has the potential to oxidize and degrade aromatic compound showing the contaminants, which are highly recalcitrant environmental pollutants. Fungal laccase-based membrane filtration, electrokinetic, adsorption, oxidation, and photocatalytic treatments are famous for PAH degradation, which steadily increases the threat to human health. Therefore, a new effective enzymatic approach in the degradation of aromatic compounds is urgently needed (Nunes and Malmlöf, 2018). Mycoremediation of PAHs in the last several years with numerous fungal species has been widely reported. A few fungal species co-metabolize the PAHs and generate a range of oxidized products (CO_2_). The fungi exhibited monooxygenase enzyme-mediated degradation of PAH (Gupta and Pathak, 2020). There are two types of fungi, ligninolytic (white-rot fungi) and non-ligninolytic fungi, which bioremediate the low and high molecular weight PAHs. Ligninolytic fungal enzymes (lignin peroxidase, manganese peroxidase, and laccases) degrade to convey the intended meaning and simultaneously oxidize the PAHs; following which they oxidize into intermediate diphenol and finally quinones (Aydin et al., 2017). Ligninolytic catalytic cleavage generates polar products (water-soluble) from aromatic compounds, which are eventually available for fungal metabolism and soil microflora in the vicinity (Gupta and Pathak, 2020). Non-ligninolytic fungi generate “cytochrome P450 monooxygenase-like” enzymes, which oxidize the PAHs; this forms arene oxide and water. After going through a non-enzymatic rearrangement process, arene oxides become phenols, which then conjugate with xylose, glucose, and gluconeric acid. (Ghosal et al., 2016). A few species produce biosurfactants in the degradation of aromatic compounds in order to overcome the hindrance of less soluble HMW PAHs (Ojha et al., 2019).

### 7.2 Mitigation of organic pollutants

A diverse range of fungal enzymes, both endocellular and exocellular, are used for waste biodegradation (Dhiman et al., 2020). Peroxidases and laccases play a role in degrading many organic contaminants. Organic pollutants in wastewater originate from various sources, including the fermentation, pulp, and paper industries. Oxidation of organic matter (such as pesticides, PCBs, PAHs, and PPCPs) occurs in the anodic chamber, while oxygen is reduced at the cathode (Ucar et al., 2017). Compounds with a high redox potential can be reduced in the cathodic chamber, such as persulfate, ferricyanide, permanganate, and nitrate (Kaneshiro et al., 2014).

### 7.3 Mitigation of wastewater pollutants

Wastewater comprises water and liquid waste from residential, commercial, industrial, and institutional sources, combined with stormwater (Adeel et al., 2018). Environmental scientists strive to remove pollution from water supplies, which often contain organic pollutants with diverse chemical structures. Polluted wastewater from numerous sources can be treated using FFCs, in which microorganisms are converted into electrical energy using chemical energy (Adeel et al., 2018). Galactomyces reessii is known to produce electricity (Chaijak et al., 2018a,b). Fungal enzymes like laccases and peroxidases aid in the degradation of pollutants to less toxic forms. Some studies employ enzyme-based techniques for complex wastewater compared to simple solutions (Martınez-Alcala et al., 2017). In FFCs, an aerobic cathodic chamber and an anaerobic anodic chamber are separated by a proton. The anodic compartment contains fermenting and microbial communities that enhance pollutant removal in the FFCs. At the anode of a fungal fuel cell, electrochemical species oxidize various substrates and organic compounds present in wastewater, producing electrons and hydrogen to reduce oxygen in the water (Li et al., 2014). Electrons are released during the degradation of organic matter. Initially, wastewater enters the anodic compartment, where fermentation of the species occurs, transforming the complex organic substances into smaller molecules (such as lactate) that are subsequently oxidized, completing the circuit (Hamza et al., 2016; Bhagchandanii et al., 2020). Some electron acceptors are fast, have high redox potential and low-cost kinetics, and are crucial for sustained environments (Lu and Li, 2012). Oxygen is the most convenient electron acceptor with a high oxidation potential, commonly used in FFCs. The availability of oxygen produces a clean product like water (Logan and Regan, 2006). Fuel cells generate electricity and help in the treatment of wastewater bodies. Fungi play a catalytic role on the anode (He et al., 2015).

### 7.4 Cathode and anode action in wastewater treatment

The cathode should be robust, mechanically strong, and highly conductive, along with catalytic properties (Mustakeem, 2015). Cathodic materials must possess high power and columbic efficiency. These electrodes play a crucial role in wastewater treatment (Rahimnejad et al., 2015; Sathishkumar et al., 2019). Cathodes can be biocathodic or abiotic, and biotic cathodes can be anaerobic or aerobic (Modestra et al., 2016). Abiotic cathodic chambers require mediators and catalysts for oxygen reduction reactions due to their high cost and susceptibility to catalyst poisoning, often seen with Pt-based materials (Liew et al., 2014). The choice of the anodic chamber is critical for the efficiency of FFCs.

#### 7.4.1 Agro-industrial wastewater treatment

Agro-industries consume substantial volumes of water during production processes, leading to the generation of wastewater rich in phosphorus, nitrogen, and organic compounds, which can be treated using FFCs. This wastewater contains oils, organic matter, grease, and proteins, contributing to the COD of surface and groundwater. Treating this wastewater can be challenging due to its resistance to bacterial degradation and significant chemical stability (Zub et al., 2008; Al-Bedak et al., 2023).

#### 7.4.2 Distillery wastewater treatment

The treatment of distillery wastewater can be achieved through natural and biological processes that facilitate the effective removal of organic pollutants. Fungal biodegradation is an environmentally friendly approach. Many states have established regulations for the wastewater produced by the agricultural industry. Aerobic degradation, particularly through fungi like white rot fungus (*Phanerochaete chrysosporium*), is efficient in removing organic matter, as indicated by Biological Oxygen Demand (BOD) and COD (Hossain, 2007). The pseudo-second kinetic model is applied in distillery wastewater treatment, and various fungal species (like *Aspergillus flavus, Fusarium verticillioides, Alternaria gaisen, Penicillium pinophilum, Aspergillus niger*, and *Pleurotus florida*), when combined, can significantly reduce COD levels (Ravikumar et al., 2011). Yeast and fungi are efficiently treated with batch aerobic treatment, leading to significant COD reduction (Hossain, 2007). Distilleries produce a significant amount of wastewater known as stillage, which poses challenges due to its low oxygen content and potential for pollution upon discharge. Distillery stillage can be employed in fungal fuel cells to enhance treatment efficiency, bioelectricity generation, and pollutant recovery. Oleaginous yeast, such as *Chlorella pyrenoidosa* and *Rhodosporidium toruloides*, effectively degrade distillery wastewater, resulting in a reduction in COD and lipid content (Ling et al., 2016). Mycelia biomass of *Calvatia gigantea* is maximized under optimal conditions when cultivated on raw distilled wastewater (Ghosh Ray and Ghangrekar, 2016). *Candida utilis* biomass can be used for wastewater treatment, resulting in a significant reduction in Dissolved Organic Carbon (DOC) (Watanabe et al., 2013). Wastewater from ethanol production contains various pollutants, and enzyme-dependent techniques, such as xylanase from phyllosphere yeast (*Pseudozyma Antarctica*), can effectively reduce DOC levels (Watanabe et al., 2015). Winery wastewater, characterized by lower organic matter concentration and high levels of polyphenolics and nutrients, contributes to water and land pollution (Melamane et al., 2007).

#### 7.4.3 Dye wastewater treatment

Dye wastewater treatment often employs physical and chemical methods, including electrochemical approaches, which are specific to dyes but can be expensive and less efficient, generating harmful intermediates (Tang et al., 2007). Alternatively, biological treatments are environmentally friendly and cost-effective for removing organic pollutants from contaminated water. Various microorganisms, including yeasts, fungi, and bacteria, are capable of decolorizing different organic substances (Qu et al., 2015). The white rot fungus *Trametes versicolor* is known for its effectiveness in treating dye wastewater (Amaral et al., 2004). Fungi can adapt to their environment and use extracellular and intracellular enzymes to metabolize and break down various dyes. Prominent examples include *Pleurotus eryngii*, *Coriolopsis* spp., and *Penicillium simplicissimum* (Chen and Yien Ting, 2015a,b). Biological processes are generally slower in decolorizing fungal strains but yield satisfactory results. These biological treatments can be applied to treat wastewater from food processing, dairies, plastics, breweries, paper production, and petrochemical industries (Abdullah et al., 2017). *Ganoderma lucidum*, an ornamental fungus, contributes to increased electricity generation in the cathodic chamber by degrading acid orange. White rot fungi like *Phanerochaete chrysosporium* are capable of degrading azo dyes through laccase activity in Fuel Cells (FCs), where the anode compartment transfers dyes to the cathode compartment (Rathi and Kumar, 2022). In FCs, laccase produced by white rot fungi is an efficient and cost-effective means of sustainable power generation, as it is generated as a byproduct of plant lignin degradation, returning nutrients to the soil. Dye wastewater decolorization occurs through the release of electrons in the anodic chamber, which are then transferred externally to the cathode. Azo dyes serve as electron acceptors and are decolorized through cathodic reactions. White rot fungi are known for their ability to completely degrade these dyes (Holkar et al., 2016).

#### 7.4.4 Pharmaceutical wastewater treatment

Wastewater from pharmaceutical plants contains active pharmaceutical compounds, leading to acute and chronic health risks due to prolonged exposure. These compounds can accumulate in tissues, inhibit cell proliferation, and lead to reproductive damage (Patneedi and Prasadu, 2015). Antibiotics in pharmaceutical wastewater cannot be effectively removed, resulting in antibiotic resistance in fungi, aquatic ecosystems, and altered microbe structures. Various treatments are used for pharmaceutical wastewater, but they often require significant space, energy, and costs. These challenges have driven researchers to explore the use of FFCs (Ismail and Habeeb, 2017).

#### 7.4.5 Treatment of heavy metals load

**Figure 5:**
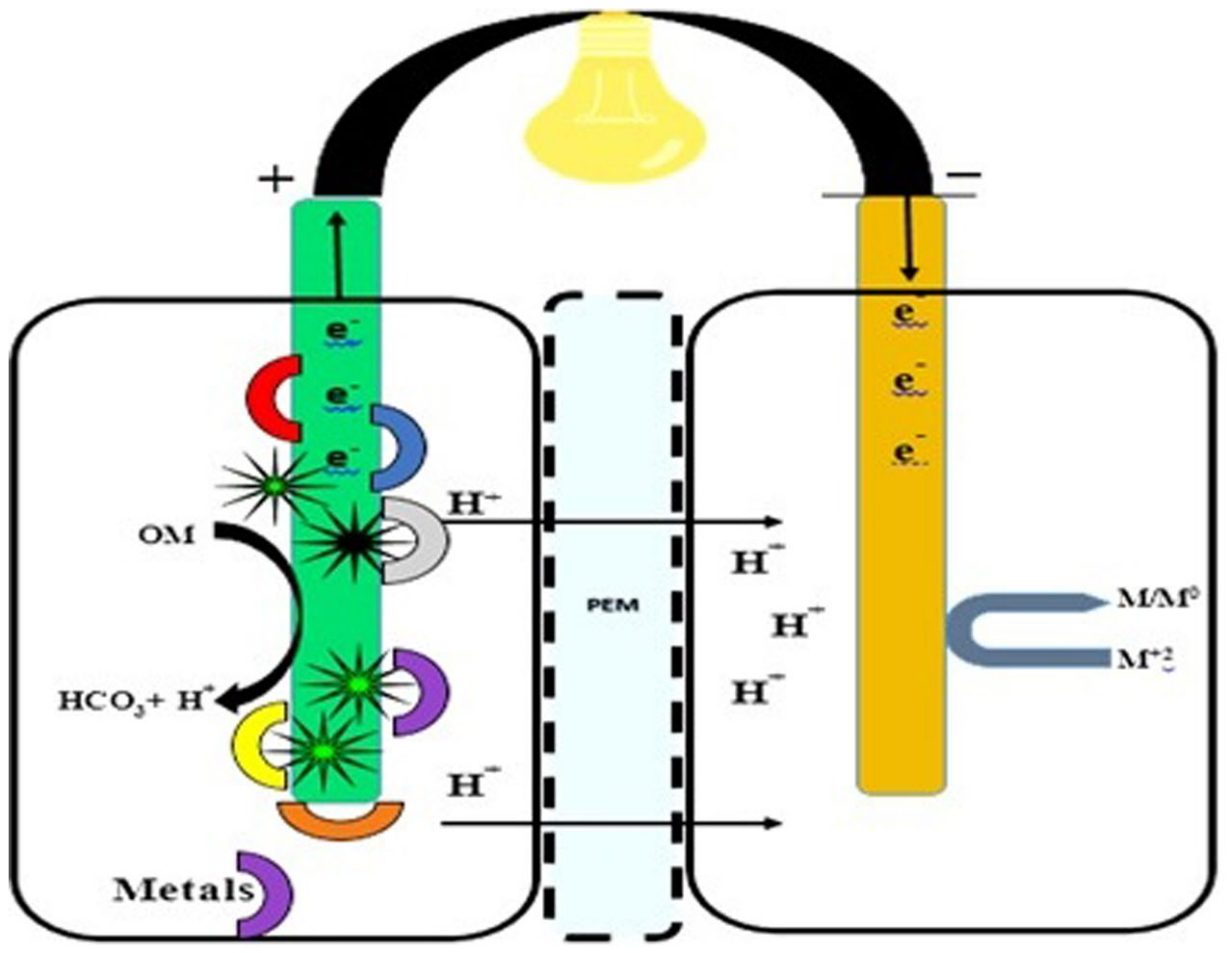
Double-Chambered fungal fuel cell for heavy metal treatment. A double-chambered fungal fuel cell designed for the treatment of heavy metal-loaded samples in water, land, and soil. Fungal mycelium or cells are attached to the anode, employing their enzymes for metal degradation.

Water, a precious resource, faces multiple forms of pollution, making traditional treatments inadequate. Innovative approaches that conserve energy and promote recovery are essential. FFCs offer a sustainable bioremediation method for treating heavy metal-containing wastewater. Both double- and single-compartment FFCs can be used to remove heavy metals (Bokhari et al., 2016). In the anodic chamber, organic matter is broken down, and heavy metals, including chromium, silver, copper, cobalt, and vanadium, are reduced in both double- and single-chambered FFCs. Heavy metals effectively serve as electron acceptors at the cathode (Nancharaiah et al., 2015). In biocathodes, the removal of heavy metals occurs through mechanisms such as bioaccumulation, bioreduction, biomineralization, and biosorption (Wu et al., 2018). Figure 5 indicates the fungal fuel cell for the mitigation of heavy metals.

##### 7.4.5.1 Cr (Chromium)

In abiotic cathodes of FFCs, chromium serves as an electron acceptor. Chromium, particularly Cr(VI), is a significant environmental contaminant that requires remediation (Peng et al., 2018). The electrochemical reduction of Cr(VI) in FFCs was first explored by Wang et al. (2008), and the adoption of a biocathode for Cr(VI) remediation was pioneered by Tandukar et al. (2009).

##### 7.4.5.2 Fe (Iron)

Iron, specifically Fe(III), is an environmentally friendly metal and an effective mediator in FFCs. Fe(III) can enhance the reduction rate of Cr(VI) through mediation mechanisms, contributing to effective remediation (Wang et al., 2017).

##### 7.4.5.3 Ag (Silver)

Silver is a natural metal widely used in various industries (Birloaga and Vegliò, 2018). Recovering silver from industrial wastewater is essential for environmental and economic benefits, as limited resources are available (Ho et al., 2018). FFCs offer a highly efficient silver recovery method with a power density of 4.25 W/m^2^ and the generation of 3.2 J of electricity from silver obtained from wastewater (Choi and Cui, 2012; Wang et al., 2013).

##### 7.4.5.4 Cu (Copper)

Copper is highly toxic and poses health hazards to living organisms, necessitating its removal from wastewater. The use of radioactive and industrial effluents for environmental protection has become crucial (Ahmed et al., 2016). FFCs can effectively remove copper, achieving a 99.88% removal rate and a high power density of 0.43 W/m^2^ in the cathodic chamber (Miran et al., 2017).

##### 7.4.5.5 Co (Cobalt)

Cobalt metal is essential for living organisms. An excess of this metal causes toxic health hazards such as asthma, dermatitis, lung cancer, and pneumonia in organisms and ecosystems (Lwalaba et al., 2020).

##### 7.4.5.6 V (Vanadium)

Almost 38,000 tons of Vanadium V(V) are produced annually throughout the world. This element is used as a steel additive and is implicated in many human diseases (Mukherjee and Chandra, 2004).

#### 7.4.6 Livestock wastewater treatment

The nitrogen and organic matter in livestock wastewater are higher, causing the production of odor. This is associated with organic acids (Castillo-Gonzalez and Bruns, 2005). In animal wastewater remedies, almost 99.76% is removed with the generation of power (Kim I. S. et al., 2008; Kim J. R. et al., 2008). Swine wastewater has expanded the feasibility of FC technology. A 115 L wastewater of pig manure was treated for 6 months via two-chambered FFCs.

#### 7.4.7 Mitigation of domestic sewage (DS)

FFCs were used to treat domestic sewage, and a huge density of power of 25 mW/m^2^ was obtained. The COD of domestic sewage has a minimal effect on the removal of organic matter but a greater effect on electricity production. A 1000 L FFC was constructed to operate the municipal sewage plant for 1 year (Rodrigo et al., 2007).

#### 7.4.8 Non-metallic (inorganic) wastewater treatment

**Figure 6:**
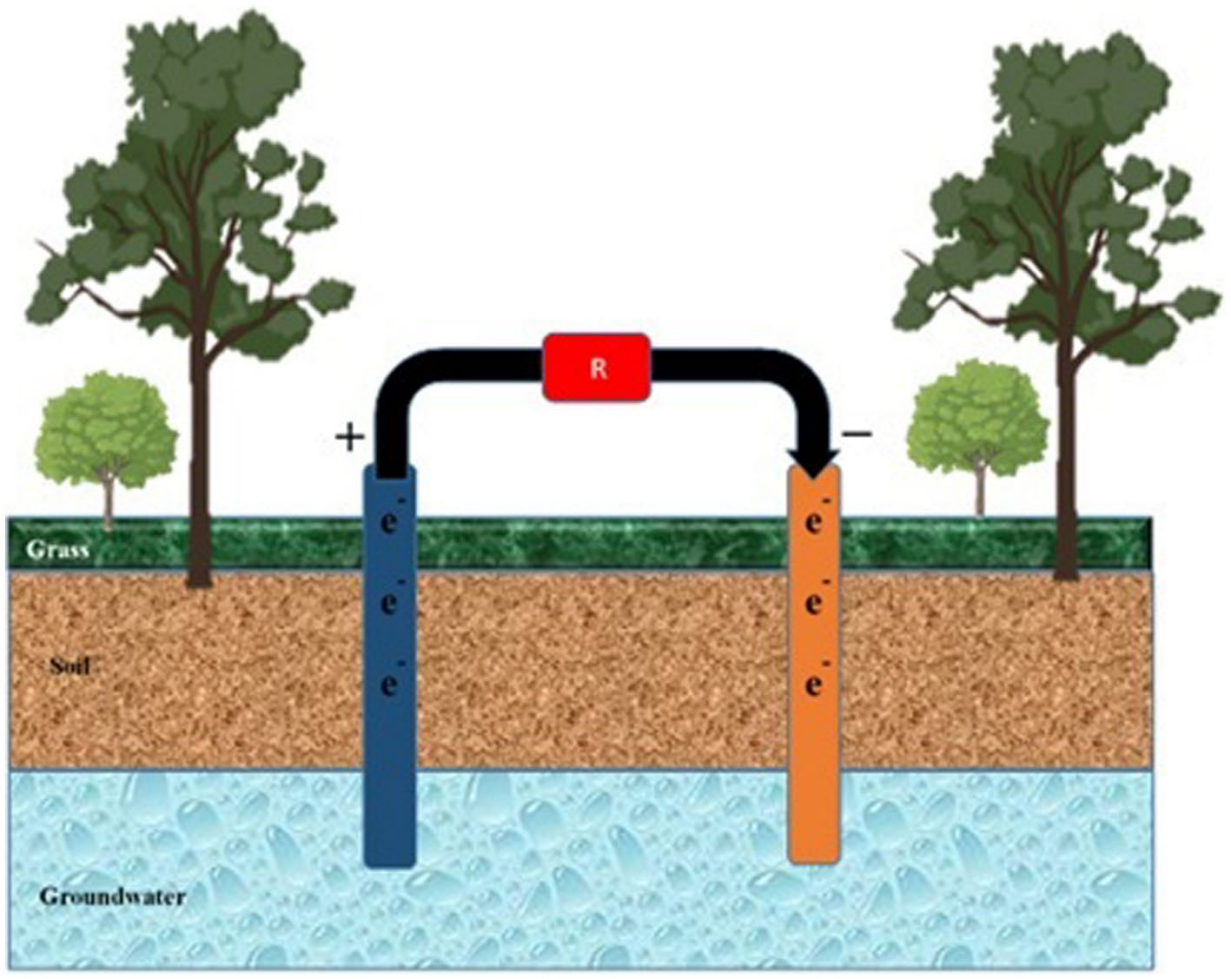
Mitigation of pollutants in groundwater. Anode and cathode components are inserted into the water from the soil surface to mitigate pollutants percolating in groundwater. ‘R’ represents the cell’s resistance to external or internal factors.

FFCs facilitate the removal of nitrates from groundwater (Figure 6). Groundwater is injected into the cathodic chamber, and acetate donates electrons from the anodic chamber (Pous et al., 2015). Sand and water are added to two compartments of the cell to enhance denitrification in a bioreactor aquifer. Nitrate loading and COD affect the removal of nitrates. The nitrate loading and COD concentration are increased (Liu et al., 2019).

#### 7.4.9 Aquatic antibiotic degradation

Conventional wastewater treatment plants contaminate the aquatic ecosystem by releasing effluents and antibiotic residues. Antibiotics, used to control diseases, are responsible for the release of thousands of tons of antibiotics each year (Carvalho and Santos, 2016). These effluents percolate into rivers and oceans (Carvalho and Santos, 2016; Lei et al., 2019), and are absorbed into the sediments (Lei et al., 2019). Antibiotic compounds are toxic to bacteria (Chen et al., 2016), aquatic species, plants, and animals (Maldonado et al., 2022). Several methods have been employed to remove antibiotic pollutants, including physical, biological, adsorption, advanced, and chemical oxidation processes (such as the Fenton process and photocatalysis) (Ajala et al., 2022). FFCs are an economical, feasible, and eco-friendly remedy for pollutant mitigation (Li et al., 2022). This technology is used along with rooted plants for the efficient removal of antibiotic residues (Bianchi et al., 2020; Wen et al., 2020).

## 8 Mitigation of land pollutants

### 8.1 Pesticides and insecticides treatment

The maximum worldwide loss of global crops is due to pests, forcing the extensive utilization of pesticides to kill the unwanted agents threatening cultured crops (Lykogianni et al., 2021). However, 90% of the pesticides do not achieve their target but instead disperse in the environment (soil systems, groundwater, atmosphere, and ocean) (Wołejko et al., 2020). Pesticides are detrimental and may cause acute lethal effects due to their high persistence rate in the environment. The following table indicates the types of pesticides degraded by fungal support. The fungal species that degraded the pesticides are listed in Table 2. The fungi causing white rot (*Pleurotus ostreatus*, *Trametes versicolor*, *Phanerochaete chrysosporium Lentinula edodes,* and *Irpex lacteus*) can disintegrate various toxic materials by their enormous reductive and oxidative reactions. Many fungi (*Fusarium oxysporum*, *Tricoderma viride, Mucor alternans,* and *Phanerochaete chrysosporium*) can degrade DDT. Endosulfan oxidizes to endosulfan sulfate by the catalysis mechanism of the fungus *Tricoderma harzianum* (Okparanma et al., 2013). Several fungi causing the degradation of polyaromatic hydrocarbons (Peng et al., 2008).

**Table 2:** Fungal mitigation action for degradation of pesticides in FFCs.

Fungal species	Pesticides	Mitigation rate	References
*Trametes versicolor*	Dicofol/Organochlorinated	87.9%	Hu et al. (2020)
	Cypermethrin/Pyrethroid	93.1%	
	Chlorpyrifos/Organophosphate	94.7%	
	Diuron and Bentazon/Herbicides	~93%	Beltrán-Flores et al. (2021)
*Ganoderma lucidum*	Diuron/Herbicide	>50%	Da Silva Coelho-Moreira et al. (2018)
*Bjerkandera adusta*	Atrazine/Herbicide	92%	
*Phanerochaete chrysosporium*	16 Organochlorinated pesticides	34.2%	Wang et al. (2014)
*Aspergillus sydowii*	Methyl parathion/Organophosphate	87–100%	Alvarenga et al. (2014)
	Chlorpyrifos	32%	Kim I. S. et al. (2008) and Kim J. R. et al. (2008)
	Methyl parathion	80%	Soares et al. (2021)
	Profenofos/Organophosphates	52%	
Maine *Aspergillus* spp.	Lambda-cyhalothrin/Pyrethroids	20.8–44.8%	Rodrigues et al. (2016)
*Penicillium decaturense, Penicillium raistrickii*	Methyl parathion/Organophosphate	87–100%	
Marine *Penicillium citrinum*	Methyl parathion/Organophosphate	100%	
*Fusarium proliferatum*	Methyl parathion/Organophosphate	100%	
Marine *Penicillium miczynskii*	Dieldrin/Organochlorinated	90%	Birolli et al. (2018)
*Acremonium* spp.,	Lambda-cyhalothrin/Pyrethroids	20.8–44.8%	
*Microsphaeropsis* spp.,	Lambda-cyhalothrin/Pyrethroids	20.8–44.8%	
*Westerdykella* spp.,	Lambda-cyhalothrin/Pyrethroids	20.8–44.8%	

### 8.2 Biodegradation of petroleum hydrocarbons (PHs)

Bioelectrochemical systems employ microbial technologies. Microorganism catalysts decompose organic compounds and generate electrons. A huge variety of pollutants in wastewater pollutes sediment and soil (Li et al., 2021; Fatehbasharzad et al., 2022). In FFCs or biotic degradation, the main mechanism is to remediate a contaminated environment. Complex organic matter is broken down into smaller molecules with the help of fungi. The chemical compounds in PHs are hardly biodegradable, including gasoline, crude oil, lubricants, diesel, and their derivatives. PHs are divided into (i) aliphatic, alkenes (alkanes and alkynes); (ii) aromatics (polycyclic aromatic and monoaromatics); (iii) cycloaliphatic (cycloalkanes); and (iv) other components (asphaltenes, waxes, resins, tar). Microbes cause the degradation of polycyclic aromatic substances dissolved in soil. These are carcinogenic compounds (Ramirez et al., 2017). The recalcitrant compounds are extremely portable in the environment, water bodies, air, and soil. PHs bioaccumulate in tissues and cause harmful effects (Poinot et al., 2014). Human-caused activities such as coal mining, municipal runoff, transportation, and storage (Varjani, 2017). *Pleurotus ostreatus* and *Irpex lacteus* degrade polyaromatic hydrocarbons from contaminated industrial soil (Bhatt et al., 2002).

### 8.3 Mitigation of plastic pollution

Plastic pollution threatens ecosystems across the globe and affects both biotic and abiotic components (Federici et al., 2022). This is a highly complex contaminant and is also the source of other contaminants. Asia and China are significant consumers and producers of goods made from plastic, commonly referred to as “white pollution.” The COVID-19 pandemic (2019) was exacerbated by the use of plastic items such as masks, tissues, gloves, and other personal protective equipment, which, when improperly disposed of as municipal waste, worsened plastic pollution (Yang et al., 2022). Plastic pollution is primarily associated with high-density polypropylene, polyethylene, and polyethylene terephthalate. The improper disposal of plastic waste has led to the dispersion of plastic pollutants on a global scale. These pollutants persist in the environment for extended periods due to their low biodegradability (Morgana et al., 2021). UV radiation can break down plastics into smaller particles, including large microplastics, small microplastics, and nano-plastics (Atugoda et al., 2022). These particles contribute to agroecosystems, soil ecosystems, marine ecosystems, and freshwater ecosystems (Chia et al., 2021; Razeghi et al., 2021). They serve as a source of carbon and electrons for microorganisms, facilitating the mineralization and degradation of pollutants (Olicón-Hernández et al., 2017). Well-known plastic-degrading species include *Aspergillus niger, Cladosporium, Penicillium simplicissimum*, and *Zalerion maritimum* (de Oliveira et al., 2020). These species utilize microplastics as a carbon source to release extracellular enzymes for degradation and promote various chemical bonding processes, leading to a decrease in their hydrophobicity. Polyurethane can be degraded by *Aspergillus fumigatus, A. tubingensis, Fusarium solani, Cladosporium pseudocladosporioides, Penicillium chrysogenum*, and *Pestalotiopsis* microspore (Magnin et al., 2020). Low-density polyethylene can be degraded by *Aspergillus favus* and *Mucor circinelloides*. The pretreatment of microplastics with nitric acid and NaOH (polyethylene) accelerates the degradation rate by *Aspergillus niger* (Kunlere et al., 2019).

### 8.4 Mitigation of soil pollutants and sedimental fuel cell

**Figure 7:**
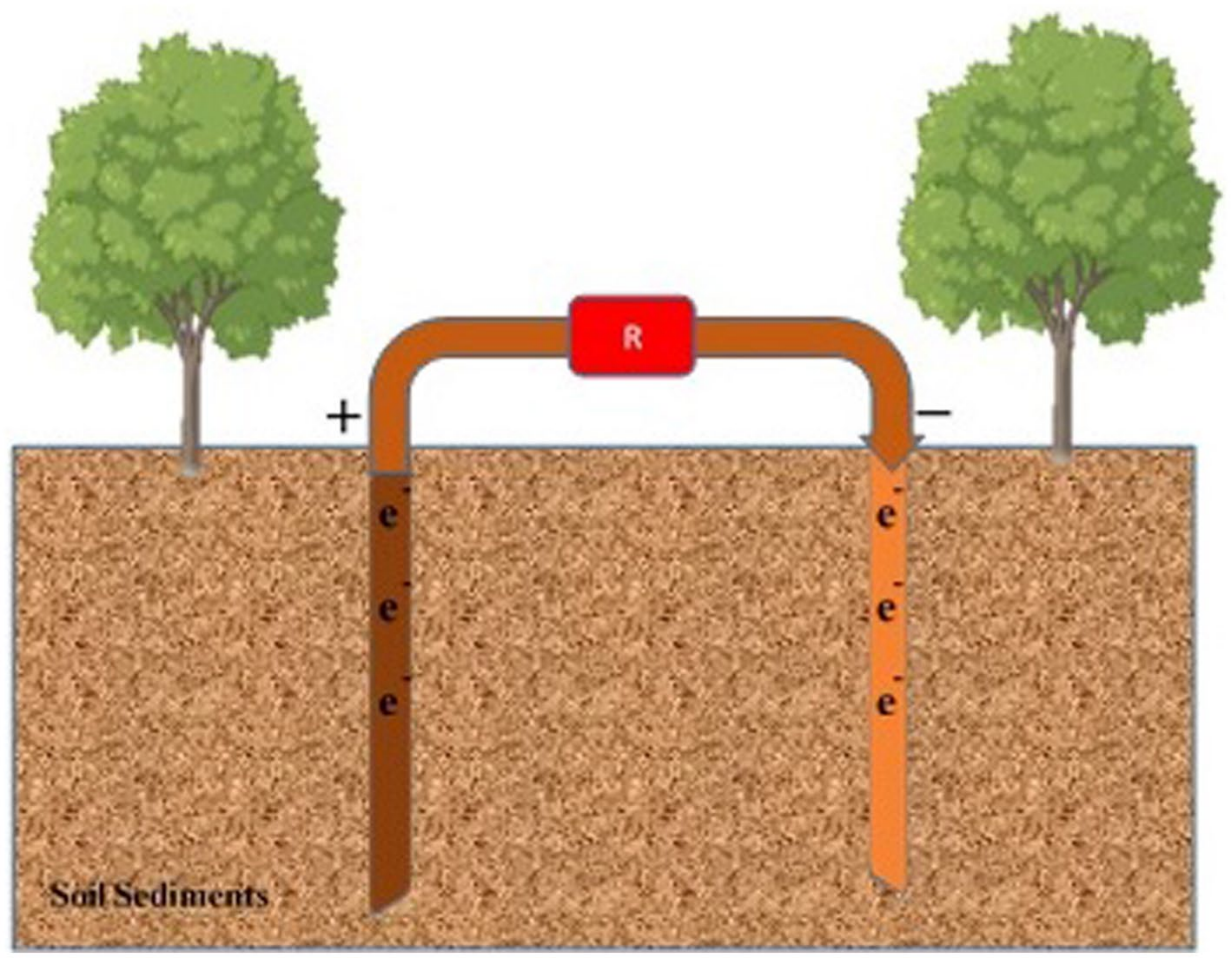
Sedimental fungal fuel cell for soil pollution mitigation. Anode and cathode components are inserted within the layers of soil sediment to mitigate soil pollutants.

Hydrocarbons in the soil can damage environmental conditions, altering soil structure and composition, leading to soil poisoning, disruptions in soil microbial communities, and hindered plant growth. Fuel cell technologies using sediment are a recent remediation technique for soil pollution. The mitigation mechanism relies on the redox gradient in both the electrodes and contaminants. Li et al. (2021) investigated the effect of glucose supplementation in sediment fuel cells for the biodegradation of pollutants in saline soil conditions, with promising results, especially in barren areas under extreme conditions (Li et al., 2016). Another competitive technology involves sediment fuel cells designed to remove hydrocarbons from the soil, with technology-based removal efficiency higher than that of contaminated sediments (Figure 7). The composition of sediment relies on various microbial populations and organic materials. Additionally, air and water quality impact the performance of fuel cell systems, helping reduce CO2 emissions and protect the environment (Sajana et al., 2017; ElMekawy et al., 2018). Mitigating organic matter in sediment fuel cells involves an anode embedded in anaerobic ingredients and an anaerobic water column cathode. This configuration aids in degrading pollutants and sediments. Improving the low conductivity of sediments for organic removal can be achieved by incorporating biochar into the sediment to enhance the removal rate of TOC and conductivity (Chen and Yien Ting, 2015a,b). Combining aquatic plants with fuel cells enhances the efficiency of organic compound removal, especially for pyrene and benzopyrene (Yan et al., 2015). Phosphorus immobilization in sediment fuel cells affects the type of phosphorus in the sediments, enhancing phosphorus stability. Treatments such as Ca-bound P, metal-bound P, and refractory P can increase P stability in the sediments (Martins et al., 2014). The anode, coated with anaerobic sediments, helps control the phosphorus content in the sediments by limiting the mixing of Fe, Al, and Ca compounds. Phosphorus is adsorbed at the expense of polyphosphate-containing organisms in the sediments (Xu et al., 2019). Mitigation of nitrogen and phosphorus removal from the sediments is crucial for maintaining the health of upper aquatic ecosystems. Fuel cells also remove heavy metals from the sediments through electrokinetics, altering the metal ions. The electric field assists in the migration of metal ions, such as Cd contents, from the sediment into the overlying water (Kabutey et al., 2019). The aerated fuel cell reduces the amount of Cu(II) to Cu(I) ions and Cr(VI) to Cr(III) ions (Abbas et al., 2018; Table 3).

**Table 3:** The role of fungal species as anode and cathode catalysts in power production.

Species	Biocatalysts fungal film	Chamber type	Power/Electricity generation	Reference
*Saccharomyces cerevisiae*	Anodic	Dual	65 mW/m^2^	Walker and Walker (2009)
*Saccharomyces cerevisiae*	Anodic	Dual	2.12 and 4.48	Permana et al. (2015)
*Saccharomyces cerevisiae*	Anodic	Dual	146.71 mW/m^3^	Gunawardena et al. (2008)
*Saccharomyces cerevisiae*	Anodic	Dual	12.9 and 20.2	Kasem et al. (2013)
*Saccharomyces cerevisiae*	Anodic	Single	80, 148, 120	
*Saccharomyces cerevisiae*	Anodic	Single	138, 344	Christwardana and Kwon (2017)
*Saccharomyces cerevisiae*	Anodic	Dual	1,500	Ganguli and Dunn (2009)
*Saccharomyces cerevisiae*	Anodic	Single	32, 39	Gal et al. (2016)
*Saccharomyces cerevisiae*	Anodic	Dual	2	Rahimnejad et al. (2011)
*Saccharomyces cerevisiae*	Anodic	Dual	33	Arbianti et al. (2012)
*Saccharomyces cerevisiae*	Anodic	Dual	123.4	Lin et al. (2017)
*Candida melibiosica*	Anodic	Dual	720	Hubenova et al. (2011)
*Arxula adeninivorans*	Anodic	Dual	1,030	Haslett et al. (2011)
*Candida melibiosica*	Anodic	Dual	20.6 mW/m^2^	Lee et al. (2015)
*Hansenula anomala*	Anodic	Dual	690–2,900 mW/m^3^	Flimban et al. (2019)
*Kluyveromyce smarxianus*	Anodic	Dual	850,000 mW/m^3^	Kaneshiro et al. (2014)
*Lipomycesstarkeyi-Klebsiellapneumoniae*	Anodic	Dual	12,870 mW/m^3^	Islam et al. (2018)
*Trametes versicolor*	Cathode	Double chamber/H-type	320 mW/m^3^	Wu et al. (2012)
*T. versicolor + S. oneidensis*	Cathode	Double chamber/H-type	1,200 mW/m^3^	Fernandez de Dios et al. (2013)
*Ganoderma lucidum*	Cathode	Single	13.38 mW/m^2^	Lai et al. (2017a,b)
*Ganoderma lucidum*	Cathode	Dual	207 mW/m^2^	Lai et al. (2017a,b)
*Galactomyces reessii*	Cathode	Dual	1,163 mW/m^3^	Chaijak et al. (2018a,b)
*Rhizopus* spp.	Cathode	Dual	317 mW/m^3^	Morant et al. (2014)
*Aspergillus* spp.	Cathode	Dual	438 mW/m^3^	
*Penicillium* spp.	Cathode	Dual	344 mW/m^3^	

## 9 Mitigation of air pollutants

### 9.1 Mitigation of gas emissions and volatile organic compounds

The main sources of CH4 emissions are wetlands and paddy fields. Fuel cells can control gas emissions using electrogenic species in the anodic chamber, which compete with organic substrates and reduce CH4 production. Fuel cells also reduce the emission of N2O and CO2 from constructed wetlands, with organic loads emitting the highest levels of CO2 and CH4 while reducing N2O emissions (Zhang et al., 2019). The incorporation of biochar into paddy fields reduces CH4 emissions, and biochar in the anodic chamber of fuel cells also leads to reduced CH4 emissions (Khudzari et al., 2019). Volatile organic compounds, such as xylene, ethylbenzene, benzene, and toluene, are degraded in fuel cells through a three-step process: (i) transfer of compounds from the gas phase to the liquid, (ii) diffusion of compounds into the biofilm, and (iii) degradation by fungi. The organic load can be reduced by the biofilm of the organism on the electrode, facilitating the rapid *in situ* electron transfer of electrons from the fungi (Kumar et al., 2019). Fungi play a more significant role in the purification performance and growth of symbiotic systems than other organisms. *Ganoderma lucidum* outperforms *Pleurotus ostreatus* and is effective in purifying biogas and biogas slurry, with removal rates of 83.94 and 68.74%, respectively (Wang et al., 2023).

### 9.2 Microbial fuel cells for environmental monitoring

FFCs have been employed in environmental monitoring, providing energy to power remote sensors. These powered biosensors are used to detect environmental pollutants in remote areas, ensuring sufficient, stable, and long-lasting power generation. However, the presence of inhibitors and toxic substances can affect the power potential of fuel cells. Self-powered fuel cell sensors are used for *in situ* online monitoring of various environmental contaminants, such as Hg, BOD, Cr, Pb, bentazon, DO, formaldehyde, and p-nitrophenol. Electroactive species serve as probes to generate electrical signals (Abrevaya et al., 2015; ElMekawy et al., 2018).

### 9.3 Degradation of miscellaneous wastes

A diverse group of fungi, including molds, yeast, and filamentous fungi, can treat industrial wastewater effectively. These fungi are capable of breaking down wood, paper, textiles, plastic, and leather. They can also degrade hydrocarbons, including polychlorinated biphenyls and phenolic compounds, using various enzymes like manganese peroxidase, laccases, and lignin peroxidases (Bhattacharya et al., 2012). Phenolic mixtures and aromatic amines are oxidized using environmental oxygen as the terminal electron acceptor (Daâssi et al., 2016).

### 9.4 Role of plants in FFCs

Plants play a crucial role in fuel cells by donating electrons to fungi and aiding in the absorption of organic contaminants. This contributes to increased solubility, immobilization, and transformation of hydrophobic pollutants. The combination of plants and fungi enhances fuel cell performance. Sand in contaminated soil promotes mass transport within fuel cells, increasing soil porosity, reducing Ohmic resistance, and improving charge output, thereby accelerating the degradation of soil hydrocarbons (Li et al., 2015). Approximately 40% of the Earth’s land is contaminated with saline-alkali soil, mainly in coastal areas due to oilfields. Salts influence soil properties, including organic matter content and metabolic activities, resulting in higher internal resistance in fuel cell performance. This issue can be mitigated by introducing carbon into the soil, which expedites the degradation process (Li et al., 2017).

### 9.5 Wetlands FCs techniques

**Figure 8:**
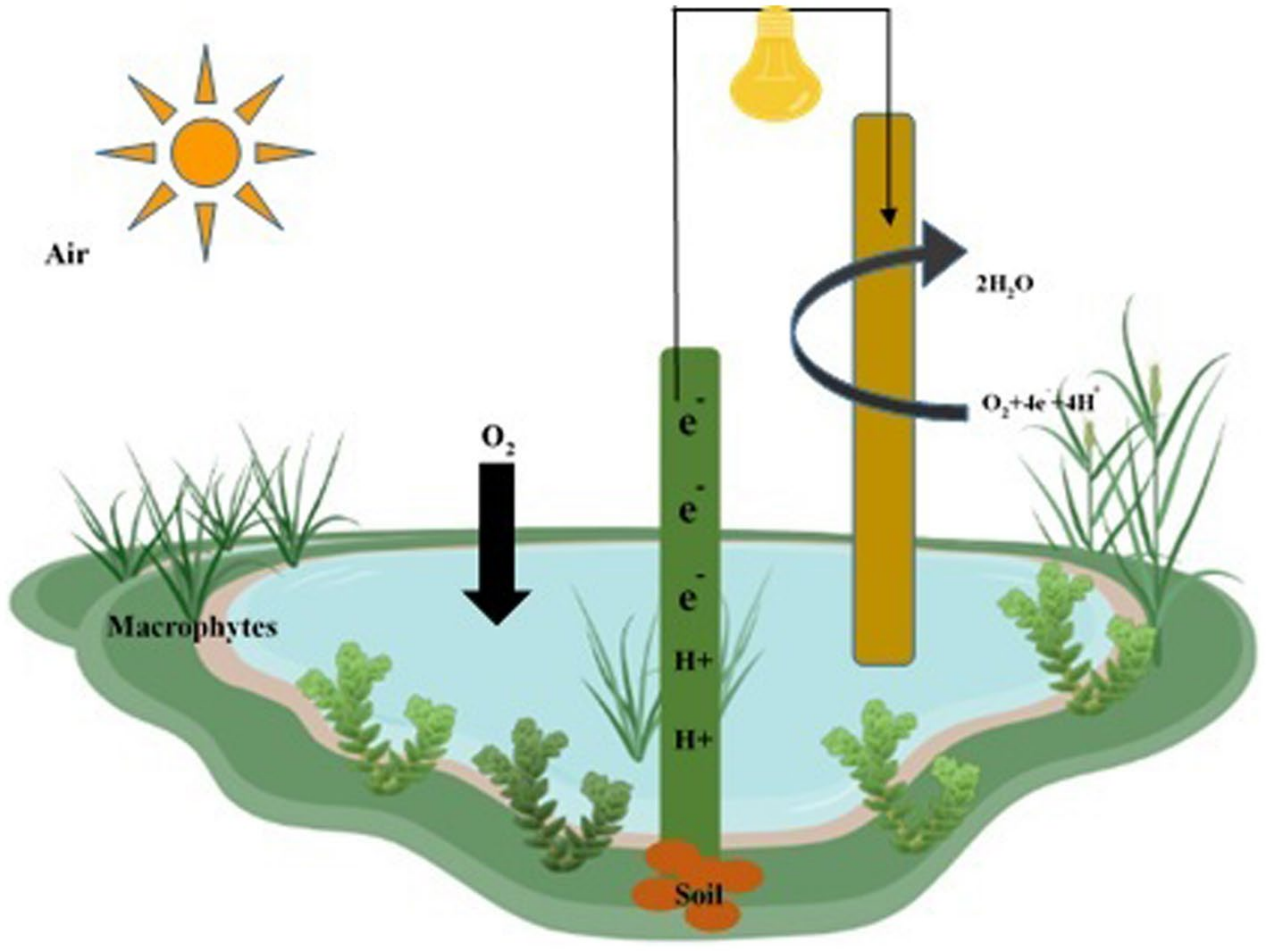
Construction of wetlands fuel cell for remediation of water, soil, and air pollutants. The anode is deeply inserted within the soil layer, while the cathode is located in the water layer to remediate water, soil, and air pollutants.

The construction of wetlands is an effective technique for removing pollutants, providing an ecologically friendly and simple approach, albeit one that requires substantial land use. Wetlands and fuel cells improve pollutant removal rates through processes like phyto-absorption and anodic zone absorption in the substrate (Wen et al., 2020). Wetland-fuel cells benefit from rooted species such as *Canna indica, Typha* spp.*, Cymbopogon citratus, Iris sibirica*, and *Oenanthe javanica*. Floating phyto species significantly enhance remediation and pollutant removal when combined with fuel cells. The design of wetlands and fuel cells incorporates macrophytes, substrates (silt and sand), and fuel cells (Figure 8), with the anode electrode placed in a zone with substrates and the cathodic zone facing the water surface. The combination of these components is instrumental in antibiotic removal. Fungi contribute to electrical production during antibiotic degradation (Wen et al., 2020). Experimental tanks are positioned horizontally, with fungi in the anodic zone, electrodes (biocathodes), antibiotics, and carbon sources enhancing electrical generation (Hassan et al., 2021). The electron transfer to fungal cells and from fungi to electrodes is facilitated by plant roots that directly contact contaminants, promoting efficient removal processes (Maldonado et al., 2022). The biotechnology components influence electricity generation and waste-to-energy conversion.

## 10 Fungal FCs in maximum power generation at anode and cathode

Fuel cells are considered sustainable and non-chemical solutions for a wide range of substrates. They operate with low power consumption, at optimum temperatures, and exhibit excellent activity. This alternative technology harnesses the potential of fungi, especially with the choice of substrates, cell configurations, anodic materials, cathodic electro-catalysts, biocatalysts, and environmental conditions (Esteve-Núñez et al., 2001). Multiple factors affect fuel cell performance, such as the choice of substrate, cell configuration, anodic material, electro-catalyst at the cathode, biocatalyst, and environmental conditions. For instance, the use of rubber sludge waste as a substrate with the bacterium *Galactomyces reessii* showed increased fungal activity in fuel cells (Chaijak et al., 2018a,b).

### 10.1 Anode catalyst

The anode plays a crucial role in fuel cells by enabling microbial electron transfer. The formation of biofilm by fungi ensures efficient electron transfer from the organism to the electrode, with different fungal species contributing to electron production from various substrates. The power output from fungal fuel cells typically ranges from mWm − 2 to several Wm − 2. It depends on factors such as anode material, cell construction, fungal species, and the use of mediators.

### 10.2 Cathode catalyst

The cathode serves as the final electron acceptor and has a significant impact on fuel cell performance. Oxygen is the primary electron acceptor, and its presence in cathodic chambers is pivotal. Oxygen reduction reactions that convert oxygen into water at the cathode can be limiting due to the overpotential of oxygen and low reaction kinetics. The use of catalysts, including enzymes, enhances the efficiency of oxygen reduction reactions at the cathode, which is essential for improved fuel cell performance (Sekrecka-Belniak and Toczyłowska-Mamińska, 2018).

## 11 Conclusion and future prospects

In conclusion, FFCs offer a versatile and innovative solution to address the global challenges of diminishing fossil fuels and environmental pollution. As the world’s population continues to grow and industrial activities expand, there is an increasing demand for alternative energy sources. FFCs provide a unique approach to harnessing bioenergy from biodegradable waste materials while actively contributing to environmental remediation. They operate through a combination of microbial and electrochemical processes. Anodes facilitate the oxidation of organic substrates, cathodes handle oxygen reduction, and proton exchange membranes conduct protons and electrons. Fungal enzymes orchestrate these essential reactions. The choice of materials for FFC electrodes is critical. Carbon-based anodes increase surface area and biocompatibility, while bio-cathodes rely on oxygen-rich chambers where fungi thrive. Fungal consortiums demonstrate remarkable potential for fine-tuning FFC performance, impacting both power generation and pollutant degradation. FFCs also open new possibilities in biofuel production, utilizing fungi to convert lignocellulosic hydrolysates into valuable lipids. Beyond energy generation, FFCs excel in pollutant removal, effectively treating various wastewater sources, including agricultural runoff, distillery effluents, and industrial wastewater containing dyes and pharmaceuticals. Fungal enzymes, such as laccases, play a crucial role in breaking down pollutants. However, a few challenges, such as scalability, cost-effectiveness, and technological optimization, are crucial for the practical implementation of FFCs. Real-world applications should be highlighted, illustrating successful case studies across diverse industries and geographical regions. Demonstrating FFCs’ efficacy in solving specific environmental problems, like wastewater treatment and pollutant removal, provides tangible evidence of their impact. Evaluating economic viability involves a thorough analysis of the costs associated with FFC development, maintenance, and scalability, juxtaposed against the benefits derived, including energy generation and environmental improvements. Identifying potential funding sources and investment opportunities can further contribute to the economic feasibility of widespread FFC adoption. Taking regulatory considerations into account is essential to navigate the complex landscape of environmental regulations and policies. Highlighting compliance with existing standards or proposing regulatory frameworks that accommodate FFC integration can facilitate their acceptance and deployment. The evolving landscape of FFCs should be explored, addressing advancements in technology, emerging trends, and potential disruptions. Assessing the global impact and prospects of FFCs involves forecasting their role in the future energy mix, their contribution to sustainable development goals, and potential collaborations with industries and governments. Overall, FFCs present a promising avenue for addressing energy needs while simultaneously mitigating pollution. Further research and development in this field hold great potential for sustainable and environmentally friendly solutions to the world’s energy and pollution challenges.

## Author contributions

AU: Conceptualization, Writing – original draft, Figure preparations. MM: Conceptualization, Writing – original draft, Figure preparations, Revision. IA: Visualization, Writing – review & editing. YI: Validation, Writing – review & editing. MAS: Collecting literature, Writing – review & editing. AS: Collecting literature, Writing – review & editing. AK: Finalization, Writing – review & editing. PKD: Collecting literature, Writing – review & editing. MKS: Project administration, Writing – review & editing. LZ: Supervision, Resources, Funding acquisition, Writing – review & editing.
